# Repairing the intestinal mucosal barrier of traditional Chinese medicine for ulcerative colitis: a review

**DOI:** 10.3389/fphar.2023.1273407

**Published:** 2023-10-24

**Authors:** Yichen Zong, Jie Meng, Tangyou Mao, Qiang Han, Peng Zhang, Lei Shi

**Affiliations:** ^1^ Second Clinical Medical College, Beijing University of Chinese Medicine, Beijing, China; ^2^ Department of Gastroenterology and Hepatology, Beijing University of Chinese Medicine Affiliated Dongfang Hospital, Beijing, China; ^3^ Department of Traditional Chinese Medicine, Health Service Center of Beiyuan Community, Beijing, China

**Keywords:** intestinal mucosal barrier, mechanism, pathogenesis, traditional Chinese medicine, ulcerative colitis

## Abstract

Damage to the intestinal mucosal barrier play an important role in the pathogenesis of ulcerative colitis (UC). Discovering the key regulators and repairing the disturbed barrier are crucial for preventing and treating UC. Traditional Chinese medicine (TCM) has been proved to be effective on treating UC and has exhibited its role in repairing the intestinal mucosal barrier. We summarized the evidence of TCM against UC by protecting and repairing the physical barrier, chemical barrier, immune barrier, and biological barrier. Mechanisms of increasing intestinal epithelial cells, tight junction proteins, and mucins, promoting intestinal stem cell proliferation, restoring the abundance of the intestinal microbiota, and modulating the innate and adaptive immunity in gut, were all involved in. Some upstream proteins and signaling pathways have been elucidated. Based on the existing problems, we suggested future studies paying attention to patients’ samples and animal models of UC and TCM syndromes, conducting rescue experiments, exploring more upstream regulators, and adopting new technical methods. We hope this review can provide a theoretical basis and novel ideas for clarifying the mechanisms of TCM against UC via repairing the intestinal mucosal barrier.

## 1 Introduction

Ulcerative colitis (UC) is a chronic inflammatory bowel disease, characterized by inflammatory ulceration of the colon and rectum. Patients with UC are troubled with persistent or recurrent diarrhea, bloody stools, and abdominal pain. In recent decades, the incidence of UC has been increasing worldwide, attracting more and more attention (Cosnes et al., 2011; Molodecky et al., 2012; Ananthakrishnan, 2015). Unfortunately, UC still cannot be cured. The aim of treatment is to induce and maintain remission, and to prevent disability, colectomy, and colorectal cancer in the long term. Based on the assessment of disease severity and extent, medications including 5-aminosalicylic acid (5-ASA) drugs, corticosteroids, immune-suppressants, and biological therapy are recommended for the treatment of UC (Harbord et al., 2017; Rubin et al., 2019). Although the early use of biological therapy, including infliximab, adalimumab, and golimumab, improves the efficacy, most medications are of various limitations, and a considerable proportion of patients still fail to achieve or maintain remission after treatment (Ungaro et al., 2017). There is an urgent need to find new therapeutic targets and new therapies for UC.

The intestinal mucosal barrier plays an important role in maintaining intestinal homeostasis and takes part in the pathogenesis of UC (Turner, 2009). It is composed of physical barrier, chemical barrier, immune barrier, and biological barrier. The physical barrier is the mainstay and prevents harmful substances from entering the intestinal mucosa, while the chemical barrier is a supplement to the physical barrier. The immune barrier prevents the damage of pathogenic antigens to the gut via the innate and adaptive immunity, and the biological barrier provides colonization resistance and helps nutrition absorption. The integrity and interaction of each barrier ensure the normal function of the intestinal mucosal barrier and the intestinal homeostasis. Early on in the pathogenesis of UC, damage to the intestinal mucosal barrier and a defective immune response to commensal bacteria might be the main mechanisms (Kobayashi et al., 2020; An et al., 2022). Mucosal injury allows the gut microbiota to trigger a sustained and uninhibited inflammatory response. Some studies also demonstrated that the intestinal barrier dysfunction could precede the clinical diagnosis of inflammatory bowel disease (IBD) by years (Torres et al., 2020; Turpin et al., 2020), which meant that discovering the key regulators and repairing the disturbed intestinal mucosal barrier would be crucial for preventing and treating UC. However, there is still a lack of medications targeting this mechanism (Ma et al., 2019; Alsoud et al., 2021; Hanzel et al., 2022).

Based on syndrome differentiation, traditional Chinese medicine (TCM) has been widely used in the treatment of digestive diseases for thousands of years. Accumulating evidences have shown that TCM has a definite therapeutic effect on UC. A multicenter, double-blind trial by Naganuma et al. found that compared with placebo, 8 weeks of Strobilanthes cusia (Nees) Kuntze [Acanthaceae; indigo naturalis] was effective in inducing a clinical response in Japanese patients with active UC (Mayo scores of 6 or more) (Naganuma et al., 2018). Shen et al. conducted a multicenter, randomized, controlled, double-blind study and demonstrated that Qing-Chang-Hua-Shi granules, a Chinese herbal formula, combined with continued 5-ASA 4 g/d therapy for 12 weeks led to a higher rates of clinical remission in moderately active UC patients (Shen et al., 2021). Another randomized controlled study showed that compared with mesalazine, Fufangkushen colon-coated capsule was similarly effective and safe in the treatment of active UC (Gong et al., 2012). Hu et al. conducted a meta-analysis and found that TCM combined with probiotics could alleviate clinical symptoms, inhibit intestinal inflammatory response, and reduce the disease recurrence with less adverse events (Hu et al., 2022). A large number of experiments *in vivo* and *vitro* also proved the efficacy of TCM formulas and metabolites on UC (Zheng et al., 2022a; Liu et al., 2022). While TCM is effective on UC, the potential mechanisms are still partially unknown. Studies have been conducted to unveil the underlying mechanisms of TCM against UC, and repairing the intestinal mucosal barrier has been reported to play an important role (Zheng et al., 2022a; Liu et al., 2022). Our team has been engaged in researches on TCM for UC for a long time, and has also found that TCM can exert therapeutic effects via repairing the intestinal physical barrier and regulating the intestinal microbiota (Mao et al., 2017; Kou et al., 2020; Sun et al., 2020; Wang et al., 2022a).

Therefore, we wrote the present review to summarize the evidence of TCM treating UC by protecting and repairing the mucosal barrier, hoping bringing new thinking to future studies.

## 2 Intestinal physical barrier

### 2.1 Basic biology of intestinal physical barrier

The intestinal physical barrier mainly consists of intestinal epithelial cells, tight junctions, and extracellular mucus (Odenwald and Turner, 2017). The intestinal epithelial cells, including goblet cells, Paneth cells, and M cells, play a central role in the intestinal physical barrier (Allaire et al., 2018). These cells can be continually renewed by pluripotent intestinal epithelial stem cells residing in the base of crypts, and some of them, including enteroendocrine cells, goblet cells and Paneth cells, are specialized for secreting mucus, AMPs, and secreted immunoglobulin A (SIgA) (Peterson and Artis, 2014). The intestinal epithelial cells can also sense and respond to microbial stimuli, and participate in the coordination of appropriate immune responses. Tight junctions, including transmembrane proteins like claudin, occludin, zonula occludens 1 (ZO-1), and cingulin, connect adjacent intestinal epithelial cells, forming the physical barrier between the apical and the basolateral plasma membrane domains (Groschwitz and Hogan, 2009). Tight junctions also contribute to the establishment of cell polarity (Cereijido et al., 1998). Mucin is secreted by goblet cells, and plays an important role in isolating intestinal mucosa from pathogenic microbes. Studies have shown that injured epithelial cells, mucus abnormalities, and disordered tight junctions all have a close association with active UC (Van der Sluis et al., 2006; Chelakkot et al., 2018).

### 2.2 Evidence on TCM formulas targeting intestinal physical barrier

Gegen Qinlian decoction, composed of Pueraria montana var. lobata [Fabaceae; kudzuvine root], Scutellaria baicalensis Georgi [Lamiaceae; scutellariae baicalensis radix], and Coptis chinensis Franch. [Ranunculaceae; coptidis rhizoma], was firstly recorded in “Treatise on Febrile Diseases”.

By means of clearing dampness and heat, it has been widely used in the treatment of diarrhea in China. Wang et al. conducted an experiment *in vivo* and showed that receiving Gegen Qinlian decoction for 6 days could alleviate dextran sulfate sodium (DSS) induced colitis in C57BL/6 mice. It also improved mucus thickness and tight junction proteins ZO-1, occludin and Claudin1, which could be reversed by administration of aryl hydrocarbon receptor (AhR) antagonists. Further experiments showed that the AhR-mediated effect of Gegen Qinlian decoction on repairing the intestinal physical barrier might be induced by regulating gut microbiota-related tryptophan metabolism and restoring the generation of indole derivatives (Wang et al., 2023). Another study *in vivo* showed that 10 days of Jiawei Gegen Qinlian decoction and the main metabolites, including puerarin, baicalein, berberine, and glycyrrhiic acid, could alleviate DSS-induced colitis in Sprague-Dawley (SD) rats and Kunming mice. The activities of Dao and D-lactate in serum were significantly decreased, and the levels of ZO-1, occludin and claudin1 in colon tissue were increased, which meant that the therapeutic effect was associated with the reduced intestinal permeability (Li et al., 2021a). Zhao et al. found that Gegen Qinlian decoction could repair the intestinal physical barrier in both acute and chronic colitis caused by DSS, which was related to the increased level of MUC2 mRNA, reduced goblet cell differentiation, and promoted intestinal stem cell proliferation. A bidirectional regulatory effect of Gegen Qinlian decoction on the Notch signal transduction might account for the above results and was verified by experiments *in vitro* and in TLR4 knockout mice (Zhao et al., 2020).

Huangqin decoction, composed of Scutellaria baicalensis Georgi [Lamiaceae; scutellariae baicalensis radix], Paeonia lactiflora Pall. [Paeoniaceae; paeoniae radix alba], Glycyrrhiza glabra L. [Fabaceae; glycyrrhizae radix et rhizoma], and Ziziphus jujuba Mill. [Rhamnaceae; jujubae fructus], was also proved to have a therapeutic effect on UC. In “Treatise on Febrile Diseases”, Huangqin decoction was recorded with clearing heat and stopping dysentery. Similar to Gegen Qinlian decoction, Huangqin decoction could alleviate DSS-induced colitis and upregulate tight junction proteins, such as claudin-1 and ZO-1 (Zheng et al., 2022b). Li et al. (2021b) found that 7 days of Huangqin decoction could alleviate DSS-induced colitis and protect the epithelial barrier integrity by inhibiting the apoptosis of epithelial cells and regulating ESR1 and PTGS2, but the study did not conduct further validation experiments. Mo et al. used DSS + high-fat diet + hot and humid environment to simulate UC with a dampness-heat syndrome in mice. Results showed that compared with Salazosulfapyridine, receiving Huangqin decoction for 7days relieved colitis and downregulated IFN-γ/JAK/ETS signaling pathway related proteins, which reduced the excessive apoptosis of the intestinal epithelial cells. No rescue experiment was conducted likewise (Mo et al., 2022).

Shaoyao decoction, originated from Huangqin decoction, is composed of Paeonia lactiflora Pall. [Paeoniaceae; paeoniae radix alba], Rheum palmatum L. [Polygonaceae; rhei radix et rhizoma], Angelica sinensis (Oliv.) Diels [Apiaceae; angelicae sinensis radix], Coptis chinensis Franch. [Ranunculaceae; coptidis rhizoma], Areca catechu L. [Arecaceae; arecae semen tostum], Dolomiaea costus (Falc.) Kasana & A.K.Pandey [Asteraceae; aucklandiae radix], Glycyrrhiza glabra L. [Fabaceae; glycyrrhizae radix et rhizoma], Scutellaria baicalensis Georgi [Lamiaceae; scutellariae baicalensis radix], and Neolitsea cassia (L.) Kosterm. [Lauraceae; cinnamomi cortex]. Receiving Shaoyao decoction for 7 days could alleviate DSS-induced colitis in Kunming mice. It was also shown to have a regulatory effect on the MKP1/NF-κB/NLRP3, which could upregulate the expression of mucin and occludin. MKP1 inhibitor could reverse the effect of Shaoyao decoction (Wei et al., 2021).

Similar mechanisms were observed in experiments on Wumei Wan, which also came from “Treatise on Febrile Diseases”. Wumei Wan consists of Prunus mume (Siebold) Siebold & Zucc. [Rosaceae; mume fructus], Coptis chinensis Franch. [Ranunculaceae; coptidis rhizoma], Phellodendron amurense Rupr. [Rutaceae; phellodendri amurensis cortex], Asarum heterotropoides F.Schmidt [Aristolochiaceae; asari radix et rhizoma], Neolitsea cassia (L.) Kosterm. [Lauraceae; cinnamomi cortex], Angelica sinensis (Oliv.) Diels [Apiaceae; angelicae sinensis radix], Zanthoxylum bungeanum Maxim. [Rutaceae; zanthoxyli pericarpium], Cyperus rotundus L. [Cyperaceae; cyperi rhizoma], Zingiber officinale Roscoe [Zingiberaceae; zingiberis rhizoma praeparatum], and Panax ginseng C.A.Mey. [Araliaceae; ginseng radix et rhizoma], and is suitable for patients with a TCM syndrome of upper heat and lower cold. Yan et al. (2022a) showed that receiving Wumei Wan for 7 days could alleviate DSS-induced acute colitis in mice, and increase the number of goblet cells and the secretion of mucus. Their another experiment showed that 15 days of Wumei Wan could relieve DSS-induced chronic colitis in mice, inhibit the apoptosis of epithelial cells, and promotes cell proliferation. Mucin 2 was also significantly upregulated. The effect of Wumei Wan on repairing the mucosal barrier might be related to the regulation of Hippo/Yes-associated protein (YAP) signaling, but there was a lack of a rescue verification (Yan et al., 2022b).

Xianglian Pill, a Chinese patent medicine, is composed of Coptis chinensis Franch. [Ranunculaceae; coptidis rhizoma] and Dolomiaea costus (Falc.) Kasana & A.K.Pandey [Asteraceae; aucklandiae radix]. It was shown to be active on alleviating DSS-induced colitis and increasing the expression of Claudin-1 and ZO-1 in the colon, which might be induced by enhancing the autophagy via blocking the activation of PI3K/Akt/mTOR signaling pathway. Adding the autophagy inhibitor 3-MA could reverse the effect of Xianglian Pill (Wang et al., 2021).

Shenling Baizhu San, a commonly used formula for diarrhea, consists of 10 Chinese herbs. Studies showed the efficacy of Shenling Baizhu San on UC (Chen et al., 2022). Treatment for 2 weeks could alleviate DSS-induced colitis and increase the expression of tight junction proteins (Liu et al., 2018).

Tongxieyaofang, firstly recorded in the Danxi’s Mastery of Medicine, is composed of Atractylodes macrocephala Koidz. [Asteraceae; atractylodis macrocephalae rhizoma], Paeonia lactiflora Pall. [Paeoniaceae; paeoniae radix alba], Saposhnikovia divaricata (Turcz. ex Ledeb.) Schischk. [Apiaceae; saposhnikoviae radix] (Fangfeng), Citrus × aurantium f. deliciosa (Ten.) M.Hiroe [Rutaceae; citri reticulatae pericarpium viride] (Chenpi). A Chinese clinical study on forty patients with UC showed that treatment of Tongxieyaofang could increase the expression of protective factors of the intestinal mucosa barrier (Yu et al., 2020).

Qingchang Wenzhong decoction, an effective herbal prescription for UC, consisted with Coptis chinensis Franch. [Ranunculaceae; coptidis rhizoma], Zingiber officinale Roscoe [Zingiberaceae; zingiberis rhizoma praeparatum], Sophora flavescens Aiton [Fabaceae; sophorae flavescentis radix], Strobilanthes cusia (Nees) Kuntze [Acanthaceae; indigo naturalis], Sanguisorba officinalis L. [Rosaceae; sanguisorbae radix], Dolomiaea costus (Falc.) Kasana & A.K.Pandey [Asteraceae; aucklandiae radix], Salvia miltiorrhiza Bunge [Lamiaceae; Salviae miltiorrhizae radix et rhizoma], and Glycyrrhiza glabra L. [Fabaceae; glycyrrhizae radix et rhizoma], and was shown to be effective in improving the intestinal permeability through upregulating the expressions of tight junction proteins and numbers of goblet cells. It could also promote the intestinal stem cells-mediated epithelial regeneration, which might be related to the activation of Wnt/β-catenin signals (Sun et al., 2021).

Strobilanthes cusia (Nees) Kuntze [Acanthaceae; indigo naturalis] appeared for the first time in a book in Tang Dynasty. It could clear dampness and heat based on TCM theory. In experiments on Strobilanthes cusia (Nees) Kuntze [Acanthaceae; indigo naturalis], treatment for 1 week could alleviate DSS-induced colitis in mice and increase the expression of E-cadherin, occludin, ZO-1, and MUC2. The mechanism of reinforced intestinal physical barrier might be associated with the effects of anti-inflammation and intestinal microbiota regulation (Yang et al., 2021).

Patrinia villosa (Thunb.) Dufr. [Caprifoliaceae; patriniae herba], firstly recorded in “ShenNongBenCaoJing”, is usually used for appendicitis, enteritis and gynecological inflammation. After the treatment of water extract of Patrinia villosa (Thunb.) Dufr. [Caprifoliaceae; patriniae herba] for 2 weeks, TNBS-induced colitis was alleviated in mice. The mucous epithelium and the goblet cells were significantly increased. The effect might be related to the anti-inflammatory effect of Patrinia villosa (Thunb.) Dufr. [Caprifoliaceae; patriniae herba] via impacting bile acid metabolism and inhibiting NF-κB signaling pathways. No rescue experiment was conducted to verify the results (Wang et al., 2022b).

Piper wallichii (Miq.) Hand.-Mazz. [Piperaceae; piper wallichii] is a Chinese herbal medicine, with the effect of dispelling wind, dredging collaterals, and promoting blood circulation. It has been used for intestinal diseases in Asia for a long time. A study showed that receiving the ethanol extract of Piper wallichii (Miq.) Hand.-Mazz. [Piperaceae; piper wallichii] for 12 days could alleviate DSS-induced colitis in mice, compared with tofacitinib. It could also repair the physical barrier by upregulating the expression of occludin, increasing cell proliferation, and inhibiting cell apoptosis. Further results suggested that the effect might be induced by inhibiting the TLR4/NF-κB/COX-2 signal pathway, while no rescue experiment was conducted (Zhao et al., 2023).

Aloe vera (L.) Burm.f. [Asphodelaceae; aloe], a Chinese herbal medicine, is usually used to treat gastrointestinal diseases such as constipation and colitis. Shi et al. found that receiving aloe vera for 10 days mitigated DSS-induced colitis and enhanced mucin expression, which correlated with decreased inflammation in gut. However, they did not further explore the upstream mechanisms underlying the correlation (Shi et al., 2021).

Another botanical drug, Astragalus mongholicus Bunge [Fabaceae; astragali radix praeparata cum melle], with the effect of tonifying Qi, was found to be effective on alleviating DSS-induced colitis, restoring the epithelial structure and mucous membrane architecture (Li et al., 2022a).

### 2.3 Evidence on TCM metabolites targeting intestinal physical barrier

Metabolites are the basis of the efficacy of TCM formulas, and researches on metabolites attract much attention. Berberine, an alkaloid extracted from some botanical drugs, has been studied for the treatment of UC. Zhu et al. showed that compared with sulphasalazin, administration of berberine hydrochloride for 6weeks could alleviate DSS-induced colitis in Wistar rats. It also significantly increased the protein expression levels of tight junctions, including occludin, claudin-1, ZO-1 and VCAM-1, which might be the results of blocking IL-6/STAT3/NF-κB signaling pathway (Zhu et al., 2019). Li et al. conducted experiments *in vivo* and *vitro,* demonstrating that berberine could upregulate the expression level of tight junction proteins and maintain a normal intestinal permeability (Li et al., 2020a; Li et al., 2020b). Wu et al. confirmed Berberine’ effects on DSS-induced colitis in cats. 7 days of Berberine could upregulate the expression of tight junction ZO-1 and occludin in colon tissue, which might be associated with the regulation of gut microbiota. No rescue experiment was conducted (Li et al., 2022b).

Indigo and indirubin are the main metabolites of Strobilanthes cusia (Nees) Kuntze [Acanthaceae; indigo naturalis]*.* Xie et al. (2023) showed that they could relieve DSS-induced colitis in Balb/C mice, and increase the expression of MUC2 and tight junction proteins.

Ginger polysaccharides, major metabolites of Zingiber officinale Roscoe [Zingiberaceae; rhizoma zingiberis recens], were also reported to be helpful in repairing the intestinal physical barrier indicated by the increased expression of occludin-1 and ZO-1 (Hao et al., 2022).

Aloe A and B are the main metabolites of Aloe vera (L.) Burm.f. [Asphodelaceae; aloe]. Shi et al. conducted experiments *in vitro* and found that receiving aloe A and B upregulated both intracellular and extracellular MUC2 expressions in LPS-stimulated LS174T cells (Shi et al., 2021).

Pulsatilla chinensis (Bunge) Regel [Ranunculaceae; pulsatillae radix], a Chinese herb for clearing heat and detoxification, is usually used for dysentery. Pulsatilla chinensis saponins are the main metabolites in Pulsatilla chinensis (Bunge) Regel [Ranunculaceae; pulsatillae radix], and are reported with anti-tumor, anti-inflammatory, anti-virus, and other pharmacological efficacies. Liu et al. found that receiving ethanolic extracts of Pulsatilla chinensis saponins for 9 days could attenuate DSS-induced colitis, increase the number of goblet cells, and reduce the injury of epithelial cells (Liu et al., 2021a).

Houttuynia cordata Thunb. [Saururaceae; houttuyniae herba] is a botanical drug in China, known for clearing heat and detoxification. Houttuynia cordata polysaccharides are abundant in Houttuynia cordata Thunb. [Saururaceae; houttuyniae herba]. Cen et al. reported that compared with Sulfasalazine, houttuynia cordata polysaccharides could relieve DSS-induced colitis *in vivo*, and increase the number of goblet cells and the expression of ZO-1 and MUC2. Experiments *in vitro* also confirmed that they could reduce the apoptosis of intestinal epithelial cells (Cen et al., 2022).

A large number of studies have explored and confirmed the effect of TCM on repairing the intestinal physical barrier, manifesting as increasing the number of colonic epithelial cells and the expression of tight junction proteins, reducing goblet cell differentiation, and promoting intestinal stem cell proliferation and mucus secretion, which is significantly related to the decreased colonic inflammation. The broad prospect of TCM against UC targeting the physical barrier can be viewed by these studies. Furthermore, some studies have explored the upstream mechanisms, which includes the regulation of signaling pathways, namely, Notch, IFN-γ/JAK/ETS, PI3K/Akt/mTOR, NF-κB, Wnt/β-catenin, and Hippo/YAP pathway, alterations of intestinal microbiota-related metabolisms, and modulation of target proteins, like AhR, ESR1, and PTGS2. Only 4 studies conducted rescue experiments to verify the effect mediated by the upstream targets (Zhao et al., 2020; Wang et al., 2021; Wei et al., 2021; Wang et al., 2023).

## 3 Repairing the intestinal chemical barrier

### 3.1 Basic biology of intestinal chemical barrier

The intestinal chemical barrier is a supplement to the intestinal physical barrier, including bile acids, digestive enzymes, lysozyme, AMPs, and mucins, *etc.* Few associated researches focused on repairing the intestinal chemical barrier. Mucins, which was mentioned in the part of the intestinal physical barrier, are the primary constituent of the mucous layer lining the gut. Mucins separate the intestinal microbiota and the intestinal epithelium, and participate in cell signaling, adhesion, growth, and immune modulation (Boltin et al., 2013). The metabolism of bile acids is another point at issue. Bile acids play an important part in maintaining the chemical barrier and regulating immune system with the gut microbiota involved, and the balance of bile acid composition was found to be disrupted in UC patients (Li et al., 2021c). Digestive enzymes and lysozymes have a bactericidal and bacteriolytic effect on microbes, while AMPs are an integral part of the innate immune system, which will be described in the part of the intestinal immune barrier (Kang et al., 2017; An et al., 2022). Colonic epithelial cells of UC patients displayed a significant increase of AMPs and lysozyme (Muniz et al., 2012).

### 3.2 Evidence on TCM targeting intestinal chemical barrier

Related researches are few. Li et al. found that Jiawei Gegen Qinlian decoction, and the main metabolites, including puerarin, baicalein, berberine, and glycyrrhiic acid, might have an effect on bile acids metabolism by a gut microbiota-dependent manner (Li et al., 2021a). Wang et al. showed the regulatory effect of Patrinia villosa (Thunb.) Dufr. [Caprifoliaceae; patriniae herba] on the bile acid metabolism via the metabolic analysis of serum and liver sample (Wang et al., 2022b). The specific mechanism of TCM on bile acids metabolism are still partially unknown.

## 4 Repairing the intestinal biological barrier

### 4.1 Basic biology of intestinal biological barrier

The intestinal biological barrier generally refers to the intestinal microbiota which comprises trillions of microorganisms. The intestinal microbiota is known as an essential “organ” of hosts, and takes part in the digestion and absorption of nutrients, promotes intestinal cell growth, prevents the colonization of pathogens, and maintains the normal immune function of the gut by releasing antimicrobial substances and improving resistance to harmful bacteria (Hu et al., 2021; An et al., 2022; Parizadeh and Arrieta, 2023). The exact composition of the intestinal microbiota is still unclear, and it is host-specific. Studies have shown that *Firmicutes* and *Bacteroides* make up 90% of the gut microbiota, and other phyla include *Actinobacteria*, *Proteobacteria*, *Fusobacteria*, and *Verrucomicrobia* (Arumugam et al., 2011). The main genera under the *Firmicutes* phylum are *Bacillus*, *Lactobacillus*, *Enterococcus*, *Clostridium*, and *Ruminococcus*, while the predominant genera in *Bacteroidota* are *Bacteroides* and *Prevotella* (Aggarwal et al., 2013). The composition of the intestinal microbiota also evolves continuously throughout the life and is susceptible to exogenous and endogenous factors (Marchesi et al., 2016). In UC patients, studies showed a reduction in the diversity of the intestinal microbiota, with decreased proportions of *Firmicutes* and increases in *Proteobacteria* (Machiels et al., 2014; Imhann et al., 2018; Nishino et al., 2018). In specific microbes, the proportion of beneficial bacteria such as the *Roseburia spp* and *lactobacillus* decreased, and the number of harmful bacteria such as *Escherichia coli*, *Bacteroides fragilis*, and *Helicobacter* increased (Jess et al., 2011; Tuci et al., 2011).

In addition, the intestinal microbiota produces short chain fatty acids (SCFAs), bile acids, and tryptophan, which are important in maintaining the intestinal immune system and the integrity of the intestinal barrier (Macfarlane and Macfarlane, 2003; Marchesi et al., 2016). Metabolic disorders caused by gut microbial imbalance can also affect the integrity of the intestinal barrier, which in turn aggravates UC.

There is also an interplay between the intestinal microbiota and intestinal epithelial cells, mucus barrier, and immune cells, which contributes to maintain the homeostasis of the intestinal microenvironment. The disturbed interaction might induce the development of UC (Fang et al., 2021).

### 4.2 Evidence on TCM formulas targeting intestinal biological barrier

Wang et al. found that Gegen Qinlian decoction restored tryptophan metabolism and regulated tryptophan metabolism-related gut microbiota. At the phylum level, Gegen Qinlian decoction could reduce *Proteobacteria* and increase the abundance of *Firmicutes* and *Bacteroidetes*. At the order level, the relative abundance of *Enterobacteriales* decreased after treatment, whereas the relative abundance of *Bacteroidales* and *Clostridiales* increased, which were considered to be the main bacterial communities metabolizing tryptophan to produce indole derivatives (Wang et al., 2023).

Zheng et al. demonstrated that Huangqin decoction could restore the abundance of the intestinal microbiota, increase the abundance of *Firmicutes,* and decrease the abundance of *Bacteroidetes*. At the genus level, *Lactobacillus* and *Bacteroidetes* significantly increased, while *Triclospira* and *Raptoidetes* significantly decreased (Zheng et al., 2022b).

Rhubarb Peony decoction, composed of Rheum palmatum L. [Polygonaceae; rhei radix et rhizoma], Paeonia × suffruticosa Andrews [Paeoniaceae; moutan cortex], (Danpi), Prunus persica (L.) Batsch [Rosaceae; persicae semen] (Taoren), Benincasa hispida (Thunb.) Cogn. [Cucurbitaceae; benincasae semen], and Natrii sulfas, was used for intestinal carbuncle in TCM. It was also reported to have an efficacy on colitis induced by DSS. Luo et al. (2019) showed that 2 weeks of Rhubarb Peony decoction promoted the growth of butyric acid-producing bacteria, namely, *Butyricicoccus pullicaecorum*, and regulated the producing of SCFA.

A prospective cohort study on Shenling Baizhu San showed that the formula combined with mesalamine could improve the diversity and abundance of the intestinal microbiota, increase the percentages of *Bacteroides*, *Blautia*, *Bifidobacterium* and *Lactobacillus* in patients with active UC, which are the major sources of tryptophan metabolites (Jiao et al., 2022).

Baitouweng decoction, composed of Pulsatilla chinensis (Bunge) Regel [Ranunculaceae; pulsatillae radix], Fraxinus chinensis subsp. rhynchophylla (Hance) A.E.Murray [Oleaceae; fraxini cortex], Coptis chinensis Franch. [Ranunculaceae; coptidis rhizoma], and Phellodendron amurense Rupr. [Rutaceae; phellodendri amurensis cortex], was firstly recorded in “Treatise on Febrile Diseases”. It is commonly used to treat dysentery or diarrhea caused by a dampness-heat syndrome. Studies found that administration of Baitouweng decoction for 7–10 days could attenuate DSS-induced colitis, increase the abundance of *Firmicutes*, *Proteobacteria, Actinobacteria, Tenericutes*, and decrease the abundance of *Bacteroidetes* (Hua et al., 2021; Xuan-Qing et al., 2021).

Sanhuangshu’ai decoction, firstly recorded in “Life-Saving Book of Classified Syndromes”, comprises of Coptis chinensis Franch. [Ranunculaceae; coptidis rhizoma], Scutellaria baicalensis Georgi [Lamiaceae; scutellariae baicalensis radix], Phellodendron amurense Rupr. [Rutaceae; phellodendri amurensis cortex], Artemisia annua L. [Asteraceae; artemisiae annuae herba]. It was shown that, after the treatment of Sanhuangshu’ai decoction for 7 days, DSS-induced colitis was alleviated, and the decrease of *Lactobacillus* and population abundance of intestinal flora caused by DSS was prevented (Wu et al., 2020).

Some experienced decoctions have also been shown to alleviate UC in clinical practice and experiments. Qingchang Wengzhong decoction was reported to have an effect on UC via a gut microbiota-dependent manner. Sun et al. showed that it could enrich the relative abundance of *Lactobacillus*, reduce pathogenic species, such as *Bacteroides* and *Streptococcus*, and enhance tryptophan metabolism. Rescue experiments were conducted and there were evidences that the effect of Qingchang Wenzhong decoction on regulating the gut microbiota could be transferred by fecal microbiota transplantation, and antibiotics could neutralize the beneficial effect (Sun et al., 2021).

Kuijieyuan decoction is a clinically validated TCM formula, used for alleviating symptoms associated with UC. It is composed of Astragalus mongholicus Bunge [Fabaceae; astragali radix praeparata cum melle], Scleromitrion diffusum (Willd.) R.J.Wang [Rubiaceae; oldenlandiae diffusae herba], Cirsium arvense var. arvense [Asteraceae; cirsii herba], Pulsatilla chinensis (Bunge) Regel [Ranunculaceae; pulsatillae radix], Prunella vulgaris L. [Lamiaceae; prunellae spica], Coptis chinensis Franch. [Ranunculaceae; coptidis rhizoma], Reynoutria japonica Houtt. [Polygonaceae; polygoni cuspidati rhizoma et radix], Atractylodes lancea (Thunb.) DC. [Asteraceae; atractylodis rhizome], and Glycyrrhiza glabra L. [Fabaceae; glycyrrhizae radix et rhizoma]. Liu et al. (2020) found that Kuijieyuan decoction increased the proportion of *Alloprevotella*, *Treponema*, *Prevotellaceae*, and *Prevotella*, and reduced the proportion of *Escherichia*, *Shigella*, and *Desulfovibrio* in colon.

Guchang Zhixie Wan is another clinically validated TCM formula produced by Shanxi institute of traditional Chinese medicine pharmaceutical factory. It consists of Prunus mume (Siebold) Siebold & Zucc. [Rosaceae; mume fructus], Zingiber officinale Roscoe [Zingiberaceae; zingiberis rhizoma praeparatum], Coptis chinensis Franch. [Ranunculaceae; coptidis rhizoma], Papaver somniferum L. [Papaveraceae; papaveris pericarpium], Dolomiaea costus (Falc.) Kasana & A.K.Pandey [Asteraceae; aucklandiae radix], and Corydalis yanhusuo (Y.H.Chou & Chun C.Hsu) W.T.Wang ex Z.Y.Su & C.Y.Wu [Papaveraceae; corydalis rhizoma]. It was evidenced that it was effective on decreasing the relative abundance of *Turicibacter* and increasing the relative abundance of *Ruminococcaceae_UCG-005* (Wang et al., 2020).

Yang et al. (2021) found that Strobilanthes cusia (Nees) Kuntze [Acanthaceae; indigo naturalis] could increase the abundance of *Lactobacillus* and decrease the abundance of *Streptococcus* and Desulfovibrio. Zingiber officinale Roscoe [Zingiberaceae; rhizoma zingiberis recens] and Panax ginseng C.A.Mey. [Araliaceae; ginseng radix et rhizoma] were also reported to increase the beneficial bacteria such as *Muribaculaceae Norank*, *Lachnospiraceae*, and *Akkermansia*, and reduce harmful bacteria such as *Bacteroides*, *Parabacteroides* and *Desulfovibrio*. They could modulate the composition and diversity of the intestinal microbiota to attenuate inflammatory responses (Wan et al., 2022). Li et al. observed that Astragalus mongholicus Bunge [Fabaceae; astragali radix praeparata cum melle] decreased the relative abundance of *Allobaculum*, *Shigella* and *Oscillospirillum*, which might be pathogenic bacteria. It significantly increased the relative abundance of *Akkermansia*, which was positively correlated with anti-inflammatory cytokines IL-10 and IgA (Zhu et al., 2019). Prunus humilis Bunge [Rosaceae; pruni semen] could increase the abundance of beneficial bacteria, including *Parasutterella, Bacteroides, Roseburia* and *Blautia* (Ran et al., 2022).

### 4.3 Evidence on TCM metabolites targeting intestinal biological barrier

Li et al. (2022b) found that berberine caused an increase in the proportion of *Lactobacillus, Prevoteaceae*, *Bifidobacteria*, and *Verrucomimicrobia*, and a decrease in the proportion of *Bacteroides* and *Proteobacteria* in DSS-induced UC mice. Wu et al. (2022) also showed an increase in the relative abundance of *Firmicutes* and *Lactobacillus*, and a decrease in *Proteobacteria* after treatment of berberine. Cen et al. (2022) found that houttuynia cordata polysaccharides increased the number of *Firmicutes* and *Bacteroides* in the intestinal microbiota, and decreased the number of *Proteobacteria*. Ginger polysaccharides could significantly reduce the abundance of *Proteobacteria*, improve the balance of *Firmicutes*/*Bacteroidetes* ratio, and increase the abundance of *Lactobacillus* and decreasing *Bacteroides* (Hao et al., 2022). Pulsatilla chinensis Saponins was effective on increasing the diversity of the gut microbiota, especially the beneficial bacteria like *norank_F_Muribaculaceae* and *norank_F_norank_O_Clostridia_UCG-014* (Liu et al., 2021a). Chlorogenic acid, a phenolic acid extracted from *Lonicera japonica* Thunb. [Caprifoliaceae; lonicerae japonicae flos], might restore the diversity of gut microbiota, reduce the abundance of *Firmicutes* and *Bacteroidetes*, and markedly increase the proportion of *Akkermansia* (Zhang et al., 2017). Combination therapy with indigo and indirubin was demonstrated to be effective on increasing the amounts of beneficial bacteria, like *norank_f_Muribaculaceae* and *Lactobacillus* (Xie et al., 2023).

Targeting gut microbiota is a hot topic in researches on UC, as well as TCM. Extensive studies provided evidences that TCM could alleviate UC via restoring the abundance of the gut microbiota, enriching the relative abundance of beneficial bacteria, reducing pathogenic species, and regulating the metabolism of bile acids, tryptophan, and butyric acid. Most studies were conducted without rescue experiments, which played a critical role in confirming these mechanisms. By transplanting the fecal microbiota of animals treated by TCM and using antibiotic therapy, the role of the gut microbiota in the effect of TCM can be verified from positive and negative perspectives. In addition, 16S rRNA sequencing has been employed in most studies to analyze the intestinal microbiota, which has a limitation of similarity within 16S for genus- and species-level differentiation (Church et al., 2020). The application of macrogenomics sequencing will contribute to screening the specific bacterial strains induced by TCM.

## 5 Repairing the intestinal immune barrier

### 5.1 Basic biology of intestinal immune barrier

The intestinal immune barrier is mainly composed of gut-associated lymphoid tissue (GALT), SIgA, and antibacterial substances such as mucins and AMPs. It is not only responsible for recognizing and eliminating pathogens, food antigens, and detrimental luminal substances, but also provides tolerance to commensal microbiota (Ren et al., 2019). GALT comprises Peyer’s lymph nodes, mesenteric lymph nodes, isolated lymphoid follicles, and scattered lymphocytes, and contains dendritic cells, T and B lymphocytes, plasma cells, innate lymphoid cells, macrophages, and neutrophils (Mörbe et al., 2021). GALT participates in recognizing and presenting antigens, and activating T and B lymphocytes to establish effective adaptive immune response (Luongo et al., 2009). SIgA, released by B cells, resides primarily on intestinal mucus layer. The main role of SIgA is to coat bacteria to form antigen-antibody complexes to neutralize the toxins produced by bacteria. AMPs play an important role in the innate immune system, which contain pathogens locally, exert an anti-inflammatory effect, and recruit immune cells. In UC patients, the dysfunction of the intestinal immune barrier contributes to the direct interactions between pathogens and the mucosal immune system, causing abnormal immune responses.

### 5.2 Evidence on TCM formula targeting intestinal immune barrier

Macrophages are key regulators of intestinal microenvironment homeostasis and can be polarized to different phenotypes in response to different signals, which mainly includes the classically activated macrophages (M1 macrophages) secreting pro-inflammatory cytokines and the alternatively activated macrophages (M2 macrophages) secreting anti-inflammatory cytokines. A large number of studies showed that TCM formulas, such as Gegen Qinlian decoction, Wumei Wan, Shenling Baizhu San, Tongxieyaofang and Qingchang Wenzhong decoction, *etc.*, could inhibit macrophage polarization towards M1 direction and promote M2 polarization. Meanwhile, they could increase the secretion of anti-inflammatory cytokines like IL-10, and decrease pro-inflammatory cytokines like IL-6, IL-1 β, and TNF- α. (Liu et al., 2021b; Yan et al., 2022a; Yan et al., 2022b; Yan et al., 2022c; Lu et al., 2022; Yu et al., 2022). The regulatory effect of Wumei Wan on macrophage was reported to be related to the regulation of p38MAPK, NF-κB and STAT6 signaling pathways (Yan et al., 2022a; Yan et al., 2022b; Yan et al., 2022c). Qingchang Wenzhong decoction might modulate M1 macrophage polarization via JAK2/STAT3 signaling pathway. Rescue experiments have not been conducted to verify the upstream mechanisms (Lu et al., 2022).

Helper T cells (Th), including Th1, Th2, Th9, and Th17, and regulatory T cells (Treg) play an important part in the UC’s pathogenesis *via* regulating the adaptive immune response. There is evidence that Th1 and Th17 can promote the development of UC, while cytokines produced by Th2 and Treg cells can inhibit the above pro-inflammation effects (Shi et al., 2011; Dominguez-Villar and Hafler, 2018; Lloyd and Snelgrove, 2018). Targeting the skewed axis of Th1/Th2 and Th17/Treg cells attracted many studies on TCM. Shenling Baizhu San, Baitouweng decoction, Rhubarb Peony decoction, Yiyi Fuzi Baijiang decoction, Kuijieling decoction and some botanical drugs, including Astragalus mongholicus Bunge [Fabaceae; astragali radix praeparata cum melle] and Piper wallichii (Miq.) Hand.-Mazz. [Piperaceae; piper wallichii], were shown to have an effect on the adaptive immune system by regulating the balance of Th17/Treg (Li et al., 2017; Yu et al., 2018; Luo et al., 2019; Miao et al., 2021; Li et al., 2022a; Qi et al., 2022; Liu et al., 2023; Xiao et al., 2023). The effect of Rhubarb Peony decoction might be related to promoting the growth of butyric acid-producing bacteria, while Yiyi Fuzi Baijiang decoction’s effect might be related to bile acid metabolites, especially 3-oxoLCA and isoalloLCA (Luo et al., 2019; Liu et al., 2023). Kuijieling might regulate the Treg/Th17 cell balance via the RA/RARα signaling pathway (Xiao et al., 2023). Astragalus mongholicus Bunge [Fabaceae; astragali radix praeparata cum melle] also showed an immunomodulatory effect on regulating the balance of Th1/Th2 cells (Zhu et al., 2019).

Wen et al. found that the group 3 innate lymphoid cells (ILC3s) and MHC II expression in mesenteric lymph nodes of UC mice were strongly suppressed and its inhibitory effect on T follicular helper cells (Tfh cells) was reduced, which led to the proliferation of Tfh cells and induced the class switching of IgA + B cells by increasing IL-4 levels, and hypersecretion of IgA. The expression of IgA levels in colon further aggravated colon mucosa. Alcoholic extracts of Yujin Powder could ameliorate UC through upregulating the proportion of ILC3s and expression of MHC II, enhancing the inhibition of ILC3s on Tfh cells, further regulating IgA response and IgA targeted colonic mucosal flora (Wen et al., 2022). Ran et al. also demonstrated that Prunus humilis Bunge [Rosaceae; pruni semen] improved the expression of SIgA related genes and further regulated intestinal mucosal immune function. There seems to be controversy about the role of TCM formulas in regulating IgA level in colon (Ran et al., 2022). While the experimental subjects were all induced by DSS, the types of mice and the different durations of experiments might be the reasons for the different results. Further researches need to be conducted to illustrate the changes of IgA level.

### 5.3 Evidence on TCM metabolites targeting intestinal immune barrier

Studies showed that berberine could alleviate DSS-induced UC by increasing the expression of SIgA, reducing the infiltration of neutrophils, dendritic cells, macrophages and NKTs, regulating the intestinal Treg/Th17 balance (Yan et al., 2012; Li et al., 2020a; Li et al., 2020b). Houttuynia cordata polysaccharides were reported to have an effect on suppressing the infiltration of macrophages and restoring the dysfunction of Th17/Treg cells (Cen et al., 2022).

Baicalin, a flavonoid isolated from Scutellaria baicalensis Georgi [Lamiaceae; scutellariae baicalensis radix], was reported to be able to decrease the proliferation of CD4^+^ CD29^+^ T cells, lead to a significant lower ratio of RORC/FOXP3 and indirectly regulate the balance of Th17/Treg differentiation *in vitro* (Yu et al., 2014).

Chlorogenic acid, might inhibit the NF- κB signaling pathway, leading to the suppression of the infiltration of macrophages, neutrophils, and CD3^+^ T cells in colon (Zatorski et al., 2015; Zhang et al., 2017).

Indirubin and isatin are metabolites of Strobilanthes cusia (Nees) Kuntze [Acanthaceae; indigo naturalis]. It was shown that indirubin alone, or combined with isatin, could significantly inhibit DSS-induced CD4 + T cell infiltration in mouse colon, and promote the generation of Foxp3 - expressing regulatory T cells (Gao et al., 2016; Gao et al., 2018). Xie et al. (2023) showed that combination of indigo and indirubin could reduce the populations of neutrophils, macrophages, and dendritic cells in the lamina propria, and significantly increase the relative quantity of CD335+CD11b+ NK cells.

Given UC is induced by the imbalance of immune homeostasis, a lot of studies focus on the impact of TCM on the intestinal immune barrier. Relevant mechanisms cover the regulation of macrophage polarization, balancing Th1/Th2 and Th17/Treg, modulating the secretion of IgA, and reducing the infiltration of neutrophils, macrophages, and dendritic cells. In some researches analyzing the proportion of immune cells, the flow cytometry analysis was replaced by detecting the related inflammatory cytokines levels or immunohistochemical analysis, which was ineligible. Besides, rescue experiments were absent from most studies. Adoptive transfer experiments on targeted immune cells, overexpressing and silencing targeted proteins should be applied to verify the logical relationship in future studies on related mechanisms. The regulatory effects of TCM on inflammatory factors is not within the scope of this review.

## 6 Conclusion and prospects

TCM, including TCM formulas and metbolites, is effective in treating UC, which has been confirmed by clinical trials and experiments. TCM usually acts on multi-targets and multi-pathways, and there is no exception in the treatment of UC. Considering the important role of the intestinal mucosal barrier in the pathogenesis of UC, our review summarized the mechanisms of TCM against UC by repairing the intestinal barrier, as shown in Tables 1, 2.

**TABLE 1:** TCM formulas alleviate UC by repairing the intestinal mucosal barrier.

TCM formula	Cellular or animal models	Main findings	Upstream mechanisms	Refers
Gegen Qinlian Decoction	DSS-induced colitis in mice	1) Repairing the physical barrier by increasing colonic goblet cells and the expression of tight junction proteins	1) Restoring the generation of indole derivatives to activate aryl hydrocarbon receptor -mediated IL-22 production	Zhao et al. (2020), Li et al. (2021a), Liu et al. (2021b), Wang et al. (2023)
		2) Repairing the immune barrier by increasing the proportion of IL-22^+^ILC3 in the lamina propria	2) Bidirectional regulation of Notch signaling pathway	
		3) Repairing the biological barrier by regulating the diversity of the gut microbiota, and significantly increasing the abundance of genes related to tryptophan metabolism		
Huangqin decoction	1) colitis caused by DSS + high-fat diet + hot and humid environment in mice	1) Repairing the physical barrier by upregulating the expression of tight junction proteins and inhibiting the apoptosis of epithelial cells	1) Regulating the expression of ESR1 and PTGS2	Li et al. (2021b), Zheng et al. (2022b), Mo et al. (2022)
	2) DSS-induced colitis in rats	2) Repairing the biological barrier by restoring the abundance of the gut microbiota, increasing the abundance of *Firmicutes,* and decreasing the abundance of *Bacteroidetes*. At the genus level, *Lactobacillus* and *Bacteroidetes* significantly increased, while *Triclospira* and *Raptoidetes* significantly decreased	2) Downregulating IFN-γ/JAK/ETS signalling pathway	
Shaoyao Decoction	DSS-induced colitis in mice	Repairing the physical barrier by reducing the apoptosis of epithelial cells and the differentiation of goblet cells, and upregulated the expression of mucin, occludin, and ZO-1	Inhibiting the MKP1/NF-κB/NLRP3 pathway	Wei et al. (2021)
Wumei Wan	DSS-induced colitis in mice	1) Repairing the physical barrier by increasing the goblet cells and the secretion of mucus, reduced the cleaved caspase-3 expression and decreased the cleaved caspase-3/caspase-3 radio	1) Regulating the Hippo/YAP signaling pathway	Yan et al. (2022a), Yan et al. (2022b), Yan et al. (2022c)
		2) Repairing the immune barrier by reducing the proportion of M1 macrophages, and promoting M2 polarization	2) Regulating the p38MAPK, NF-κB and STAT6 signaling pathways	
Xianglian Pill	DSS-induced colitis in mice	Repairing the physical barrier by increasing the expression of Claudin-1 and ZO-1 in the colon	Enhancing the autophagy *via* blocking the activation of PI3K/Akt/mTOR signaling pathway	Wang et al. (2021)
Shenling Baizhu San	DSS-induced colitis in mice	1) Repairing the physical barrier by increasing the expression of tight junction proteins		Liu et al. (2018), Yu et al. (2018), Jiao et al. (2022), Qi et al. (2022), Yu et al. (2022)
		2) Repairing the biological barrier by improving the diversity and abundance of the intestinal microbiota, increasing the percentages of *Bacteroides*, *Blautia*, *Bifidobacterium* and *Lactobacillus*, which are the major sources of tryptophan metabolites		
		3) Repairing the immune barrier by inhibiting the macrophage polarization towards M1 direction, and regulating the balance of Th17/Treg		
Tongxieyaofang	patients with UC	Repairing the physical barrier by increasing the expression of protective factors of the intestinal mucosa barrier, including occludin, claudin-1, and β-defensin		Yu et al. (2020)
Qingchang Wengzhong decoction	DSS-induced colitis in mice	1) Repairing the physical barrier by upregulating the expressions of tight junction proteins and numbers of goblet cells, and promoting the intestinal stem cells-mediated epithelial regeneration	1) Activating the Wnt/β-catenin pathway	Sun et al. (2021), Lu et al. (2022)
		2) Repairing the biological barrier by enriching the relative abundance of *Lactobacillus*, reducing pathogenic species, and enhancing tryptophan metabolism	2) Regulating he JAK2/STAT3 pathway	
Rhubarb Peony decoction	DSS-induced colitis in mice	1) Repairing the biological barrier by restoring the abundance of gut microbiota, increasing significantly the abundance of Firmicutes and Actinobacteria, and decreasing the Proteobacteria and Bacteroidetes	Promoting the growth of butyric acid-producing bacteria, namely, Butyricicoccus pullicaecorum	Luo et al. (2019)
		2) Repairing the immune barrier by regulating the balance of Th17/Treg		
Baitou Weng decoction	DSS-induced colitis in mice	1) Repairing the biological barrier by increasing the abundance of *Firmicutes*, *Proteobacteria, Actinobacteria, Tenericutes*, and decreasing the abundance of *Bacteroidetes*		Hua et al. (2021), Miao et al. (2021), Xuan-Qing et al. (2021)
		2) Repairing the immune barrier by regulating the balance of Th17/Treg		
Sanhuangshu’ai decoction	DSS-induced colitis in mice	Repairing the biological barrier by preventing the decrease of *Lactobacillus* and population abundance of intestinal flora		Wu et al. (2020)
Kuijieyuan decoction	DSS-induced colitis in rats	Repairing the biological barrier by increasing the proportion of *Alloprevotella*, *Treponema*, *Prevotellaceae*, and *Prevotella*, and reducing the proportion of *Escherichia*, *Shigella*, and *Desulfovibrio* in colon		Liu et al. (2020)
Guchang Zhixie Wan	DSS-induced colitis in mice	Repairing the biological barrier by decreasing the relative abundance of *Turicibacter* and increasing the relative abundance of *Ruminococcaceae_UCG-005*		Wang et al. (2020)
Yiyi Fuzi Baijiang decoction	TNBS-induced colitis in rats	Repairing the immune barrier by regulating the balance of Th17/Treg cells		Liu et al. (2023)
Kuijieling decoction	TNBS-induced colitis in rats	Repairing the immune barrier by regulating the balance of Th17/Treg cells	Inhibiting expression of IL-6R and IL-23R genes and production of RORγt	Xiao et al. (2023)
Yujin Powder	DSS-induced colitis in mice	Repairing the immune barrier by upregulating the proportion of ILC3s and expression of MHC II, enhancing the inhibition of ILC3s on Tfh cells, further regulating IgA response and IgA targeted colonic mucosal flora		Wen et al. (2022)
Strobilanthes cusia (Nees) Kuntze [Acanthaceae; indigo naturalis]	DSS-induced colitis in mice	1) Repairing the physical barrier by increasing the expression of E-cadherin, occludin, ZO-1, and MUC2		Yang et al. (2021)
		2) Repairing the biological barrier by increasing the abundance of *Lactobacillus* and decreasing the abundance of *Streptococcus* and Desulfovibrio		
Patrinia villosa (Thunb.) Dufr. [Caprifoliaceae; patriniae herba]	TNBS-induced colitis in rats	1) Repairing the physical barrier by increasing the mucous epithelium and numbers of goblet cells		Wang et al. (2022b)
		2) Repairing the chemical barrier by regulating the bile acid metabolism		
Piper wallichii (Miq.) Hand.-Mazz. [Piperaceae; piper wallichii]	DSS-induced colitis in mice	1) Repairing the physical barrier by inhibiting the apoptosis of colonic epithelial cells, and increasing the number of goblet cells and tight junction proteins		Zhao et al. (2023)
		2) Repairing the immune barrier by regulating the balance of Th17/Treg		
Aloe vera (L.) Burm.f. [Asphodelaceae; aloe]	DSS-induced colitis in rats	Repairing the physical barrier by enhancing mucin expression		Shi et al. (2021)
Astragalus mongholicus Bunge [Fabaceae; astragali radix praeparata cum melle]	DSS-induced colitis in mice	1) Repairing the physical barrier by inhibiting the apoptosis of colonic epithelial cells, and increasing tight junction proteins and mucous secreting proteins		Li et al. (2022a)
		2) Repairing the biological barrier by decreasing the relative abundance of Allobaculum, *Shigella* and Oscillospirillum, and increasing the relative abundance of Akkermansia		
		3) Repairing the immune barrier by regulating the balance of Th17/Treg and Th1/Th2		
Zingiber officinale Roscoe [Zingiberaceae; rhizoma zingiberis recens] and Panax ginseng C.A.Mey. [Araliaceae; ginseng radix et rhizoma]	DSS-induced colitis in mice	Repairing the biological barrier by increasing the beneficial bacteria such as *Muribaculaceae Norank*, *Lachnospiraceae*, and *Akkermansia*, and reducing harmful bacteria such as *Bacteroides*, *Parabacteroides* and *Desulfovibrio*		Wan et al. (2022)
Prunus humilis Bunge [Rosaceae; pruni semen]	DSS-induced colitis in mice	1) Repairing the biological barrier by increasing the abundance of beneficial bacteria, including *Parasutterella, Bacteroides, Roseburia* and *Blautia*		Ran et al. (2022)
		2) Repairing the immune barrier by improving the expression of SIgA related genes		

DSS, dextran sulfate sodium; TNBS, 2,4,6-trinitro-benzene sulfonic acid.

**TABLE 2:** TCM metabolites alleviate UC by repairing the intestinal mucosal barrier.

TCM metabolites	Cellular or animal models	Main findings	Upstream mechanisms	Refers
Berberine	1) DSS-induced colitis in rats	1) Repairing the physical barrier by increasing the expression of occludin, claudin-1, ZO-1 and VCAM-1, and promoting the differentiation of ISCs	Modulating gut enteric glial cells– intestinal epithelial cells –immune cell interactions	Yan et al. (2012), Zhu et al. (2019), Li et al. (2020a), Li et al. (2020b), Li et al. (2022b), Wu et al. (2022)
	2) DSS-induced colitis in mice	2) Repairing the biological barrier by increasing the proportion of *Lactobacillus*, Prevoteaceae, Bifidobacteria, and Verrucomimicrobia, and decreasing the proportion of *Bacteroides* and Proteobacteria		
	3) DSS-induced colitis in cats	3) Repairing the immune barrier by increasing the expression of SIgA, reduced the infiltration of neutrophils, dendritic cells, macrophages, and NKTs, regulating the intestinal Treg/Th17 balance, and reversing the upregulation of IL-17 secretion by CD4 + cells in spleen and mesenteric lymph node cells		
	4) Cytokines-primed human epithelial cell lines, HT-29 and Caco-2 cells			
Indigo and indirubin	DSS-induced colitis in mice	1) Repairing the physical barrier by increasing the level of ZO-1, occludin, and E-cadherin		Xie et al. (2023)
		2) Repairing the biological barrier by increasing the amounts of beneficial bacteria, like norank_f_Muribaculaceae and *Lactobacillus*		
		3) Repairing the immune barrier by reducing the populations of neutrophils, macrophages, and dendritic cells in the lamina propria, and increasing the relative quantity of the CD335+ CD11b+ NK cells		
Ginger polysaccharides	DSS-induced colitis in mice	1) Repairing the physical barrier by increasing the expression of ZO-1 and occludin-1		Hao et al. (2022)
		2) Repairing the biological barrier by reducing the abundance of Proteobacteria, and improving the balance of Firmicutes/Bacteroidetes ratio		
Aloe A and aloe B	DSS-induced colitis in rats	Repairing the physical barrier by enhancing mucin expression, promoting colonic mucus secretion, increasing the thickness of the colonic mucus layer, and reducing the contact of the colonic wall with bacteria and other harmful substances		Shi et al. (2021)
Pulsatilla chinensis saponins	DSS-induced colitis in rats	1) Repairing the physical barrier by increasing the number of goblet cells, improving the colonic mucosa structure, and reducing the injury of epithelial cells		Liu et al. (2021a)
		2) Repairing the biological barrier by increasing the diversity of the gut microbiota, especially the beneficial bacteria like norank_F_Muribaculaceae and norank_F_norank_O_Clostridia_UCG-014		
Houttuynia cordata polysaccharides	1) DSS-induced colitis in mice	1) Repairing the physiical barrier by reducing the apoptosis of intestinal epithelial cells *in vivo* and *in vitro*, and increasing the number of goblet cells and the expression of ZO-1 and MUC2		Cen et al. (2022)
	2) IEC-6 cells	2) Repairing the biological barrier by increasing the number of Firmicutes and *Bacteroides* and decreased the number of Proteobacteria		
	3) Ana-1 macrophages	3) Repairing the immune barrier by suppressing the infiltration of macrophages and restoring the dysfunction of Th17/Treg cells		
Chlorogenic acid	1) DSS-induced colitis in mice	1) Repairing the biological barrier by restoring the diversity of gut microbiota, reducing the abundance of Firmicutes and Bacteroidetes, and markedly increasing the proportion of Akkermansia	Inhibiting the NF-κB signaling pathway	Zatorski et al. (2015), Zhang et al. (2017)
	2) TNBS-induced colitis in rats	2) Repairing the immune barrier by suppressing the infiltration of macrophages, neutrophils, and CD3^+^ T cells in colon		
Baicalin	Ulcerative colitis patients	Repairing the immune barrier by decreasing the proliferation of CD4^+^ CD29^+^ T cells, leading to a significant lower ratio of RORC/FOXP3 and indirectly regulating the balance of Th17/Treg differentiation *in vitro*		Yu et al. (2014)
Indirubin and isatin	DSS-induced colitis in mice	Repairing the immune barrier by reducing CD4 + T cell infiltration in mouse colon, and promoting the generation of Foxp3–expressing regulatory T cells		Gao et al. (2016), Gao et al. (2018)

DSS, dextran sulfate sodium; TNBS, 2,4,6-trinitro-benzene sulfonic acid.

**FIGURE 1:**
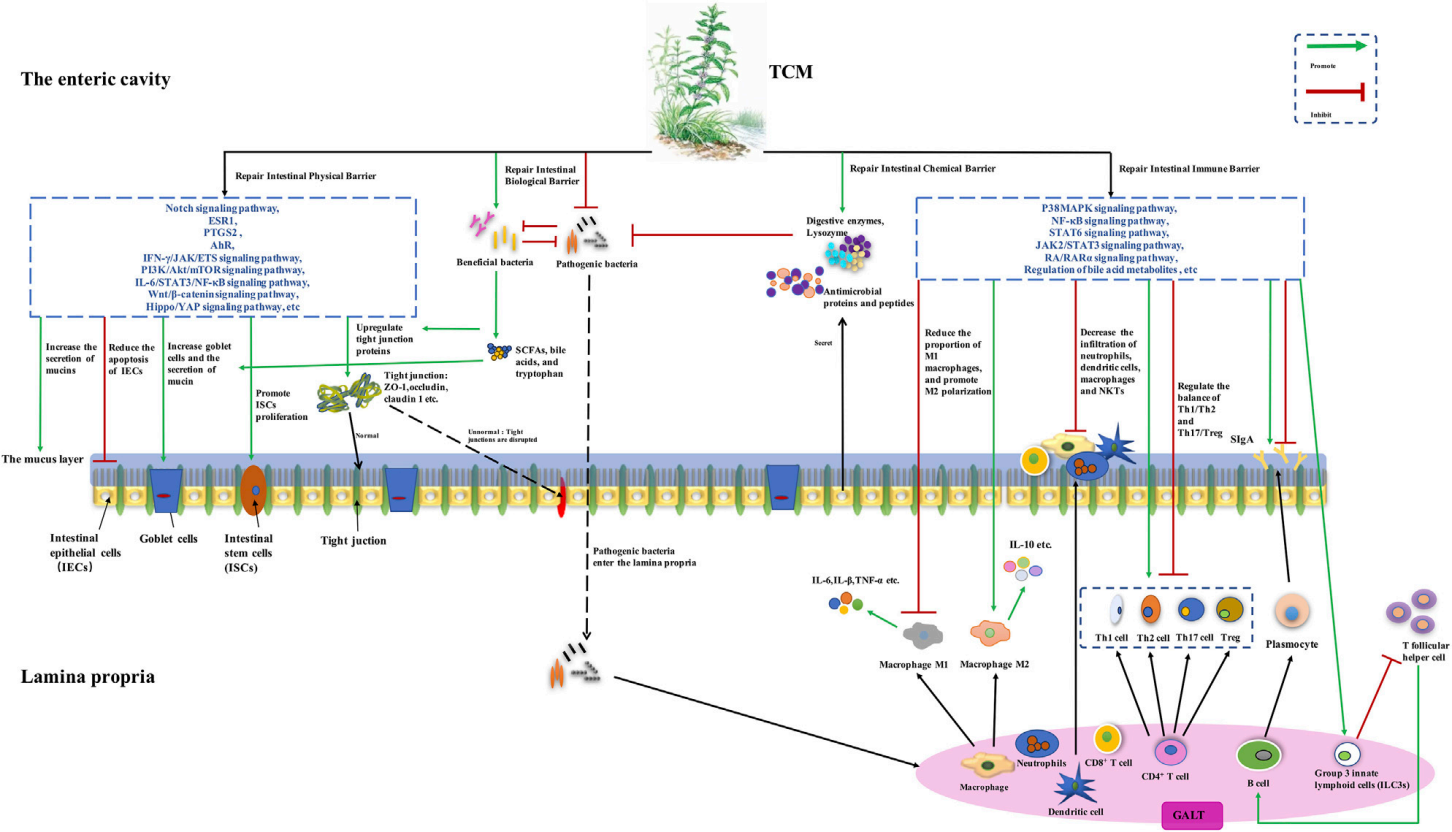
The potential mechanisms of traditional Chinese medicine (TCM) for ulcerative colitis (UC) based on repairing the intestinal mucosal barrier. TCM may increase the number of colonic goblet cells and the expression of tight junction proteins, reduce goblet cell differentiation, and promote intestinal stem cell proliferation and mucus secretion, via modulating signal pathways, including Notch, IFN-γ/JAK/ETS, PI3K/Akt/mTOR, NF-κB, Wnt/β-catenin, Hippo/YAP, AhR, ESR1, and PTGS2. The abundance of the intestinal microbiota is restored, with the relative abundance of beneficial bacteria being enriched and pathogenic species being reduced. The metabolisms of bile acids, tryptophan, and butyric acid are regulated. Macrophage polarization, balancing Th1/Th2 and Th17/Treg, changing the secretion of IgA, and reducing the infiltration of neutrophils, macrophages, and dendritic cells, were also involved in repairing the intestinal immune barrier, and modulating p38MAPK, NF-κB, STAT6, JAK2/STAT3, and RA/RARα pathways, and regulation of bile acid metabolites were found to be the upstream mechanisms.

There is compelling evidence that TCM formulas and metabolites exert an effect on repairing the intestinal physical barrier by increasing colonic epithelial cells, tight junction proteins, and mucus secretion, and promoting intestinal stem cell proliferation. The biological barrier was repaired via restoring the abundance of the intestinal microbiota, enriching beneficial bacteria, reducing pathogenic species, and regulating microbial metabolites. *Firmicutes*, *Bacteroidetes*, *Actinobacteria, Tenericutes*, *Bacteroidales*, *Clostridiales*, *Lactobacillus*, *Bacteroides*, *Blautia*, *Bifidobacterium, Alloprevotella*, *Treponema*, *Prevotella*, *Ruminococcaceae_UCG-005*, *Muribaculaceae Norank*, *Lachnospiraceae*, *Akkermansia*, *Parasutterella*, *Roseburia, Prevoteaceae*, *Bifidobacteria*, *norank_F_Muribaculaceae*, and *norank_F_norank_O_Clostridia_UCG-014* might be beneficial microorganisms, and *Proteobacteria*, *Bacteroidetes*, *Enterobacteriales*, *Triclospira*, *Raptoidetes*, *Streptococcus*, *Escherichia*, *Desulfovibrio*, *Turicibacter*, *Parabacteroides*, *Allobaculum*, *Shigella*, and *Oscillospirillum* were reported as potential harmful microorganisms. The immune barrier was restored due to the regulation of macrophage polarization, Th1/Th2 and Th17/Treg cells balance, the secretion of IgA, and reducing the infiltration of pro-inflammatory cells. Studies targeting the intestinal chemical barrier are few. Upstream mechanisms were explored, and the regulation of signal pathways, including Notch, IFN-γ/JAK/ETS, PI3K/Akt/mTOR, NF-κB, Wnt/β-catenin, Hippo/YAP, p38MAPK, STAT6, JAK2/STAT3, and RA/RARα pathways, alterations of intestinal microbiota-related metabolisms, and modulation of target proteins, like AhR, ESR1, and PTGS2, were involved. Detailed information was drawn in Figure 1.

However, attention should be paid to some problems. Firstly, most studies of TCM adopted DSS or TNBS induced colitis models. On one hand, studies on UC patients are few. On the other hand, since the treatment of TCM is based on the syndrome differentiation, new animal models simultaneously imitating diseases and syndromes deserve exploring. Secondly, there is a lack of rescue experiments in most researches, which results in incredibility and flaws in study results. For example, the impact of TCM on the gut microbiota can be verified through fecal bacteria transplantation and antibiotic therapy. There is also a scarcity of overexpression or silencing experiments of targeted proteins and signaling pathways. Meanwhile, the underlying upstream mechanisms require more elucidation. Last but not least, new technical methods, like multi omics and single-cell sequencing, will contribute to the discovery of new mechanisms.

In conclusion, we have summarized evidences in TCM formulas and metabolites, which can ameliorate experimental colitis by repairing the intestinal mucosal barrier. Increasing intestinal epithelial cells, tight junction proteins, and mucins, promoting intestinal stem cell proliferation, upregulating the abundance of the intestinal microbiota, especially beneficial bacteria, and modulating the innate and adaptive immunity in gut, were all involved in. Some targeted proteins and signaling pathways have been found to be the upstream mechanisms. Considering potential problems, suggestions including using patients’ samples and animal models of UC and TCM syndromes, conducting rescue experiments, exploring upstream mechanisms, and application of new technical methods have been put forward. Our review will provide a theoretical basis and novel ideas for future studies on clarifying the mechanisms of TCM against UC via repairing the intestinal mucosal barrier.
